# Association of objectively measured physical activity with phase angle obtained from bioelectrical impedance analysis in older adults with disabilities under the long-term care insurance system

**DOI:** 10.1186/s40101-025-00416-4

**Published:** 2025-11-29

**Authors:** Yoshihiro Fukumoto, Masanori Wakida, Ryo Kubota, Shinobu Yamazaki, Tsuyoshi Asai, Masashi Taniguchi, Jiro Nakano, Haruhiko Sato, Kimitaka Hase

**Affiliations:** 1 Faculty of Rehabilitation, Kansai Medical University, Hirakata, Osaka 573-1136 Japan; 2 KMU Day-Care Center Kori, Kansai Medical University Kori Hospital, Neyagawa, Osaka 572-8551 Japan; 3 Department of Rehabilitation, Kansai Medical University Medical Center, Moriguchi, Osaka 570-8507 Japan; 4 Human Health Sciences, Graduate School of Medicine, Kyoto University, Kyoto, 606-8507 Japan; 5 Department of Physical Medicine and Rehabilitation, Kansai Medical University, Hirakata, Osaka, 573-1010 Japan

**Keywords:** Phase angle, Physical activity, Long-term care, Bioelectrical impedance analysis, Accelerometer, Skeletal muscle mass index, Muscle quality

## Abstract

**Aim:**

The phase angle (PhA), assessed using bioelectrical impedance analysis (BIA), is becoming increasingly popular as an index of muscle quality associated with various health-related outcomes. This study aimed to clarify the relationship between PhA and sedentary behavior (SB), light physical activity (LPA), and moderate-to-vigorous physical activity (MVPA), which were objectively measured using accelerometers in older adults with disabilities requiring care.

**Methods:**

We recruited 90 older adults (39 men and 51 women, mean age of 78.7 ± 6.7 years) with disabilities under the long-term care insurance system. Skeletal muscle mass index (SMI) and PhA of the lower limbs were measured using a multifrequency BIA instrument. Daily durations of SB, LPA, and MVPA per day were measured using a triaxial accelerometer. Nutritional status was assessed using the long form of the Mini Nutritional Assessment (MNA).

**Results:**

The MVPA duration was significantly associated with lower limb PhA after adjusting for age, sex, SB and LPA durations, MNA score, and medical history (*p* = 0.037), whereas SB and LPA durations were not associated with lower limb PhA. The duration of SB, LPA, and MVPA were not significantly associated with lower limb SMI, whereas the MNA score was.

**Conclusions:**

Lower limb PhA, but not lower limb SMI, was associated with MVPA duration, independent of nutritional status and medical history. Enhancing the duration of MVPA is needed to maintain the PhA and prevent further decline in physical function in older adults who require long-term care due to disabilities.

## Background

Due to aging of society, the increase in the number of older adults who have disabilities in activities of daily living and require long-term care is a serious concern. To prevent a further decline in the ability of older adults to perform activities of daily living, maintenance of their health and physical function is important.

Muscle properties, such as muscle mass and quality, play critical roles in physical function and activities of daily living. Bioelectrical impedance analysis (BIA) is a convenient, low-cost, and noninvasive tool for measuring body and muscle composition. The skeletal muscle mass index (SMI), which is the appendicular skeletal muscle mass (ASM) of the four limbs divided by the square of the height, is a common indicator of muscle mass obtained from BIA. Recently, the phase angle (PhA) assessed using BIA has also become increasingly popular as an index of muscle quality. Bioelectrical impedance analysis consists of two impedance components: resistance (R) and reactance (Xc), and PhA is directly obtained from the relationship between R and Xc [1]. The PhA has been interpreted as an indicator of cellular health, including cell membrane integrity and function [2, 3]. A lower PhA is associated with lower muscle mass and strength [2, 4, 5], poor physical function [2, 6], incident disability [7], and mortality [8]. Because the PhA is obtained from raw impedance data without equations or models of body composition, it is not prone to equation-inherent errors [2, 9]. Thus, PhA is increasingly utilized as a surrogate measure of muscle quality in research and clinical settings.

Physical activity is one of the factors that influences health-related outcomes, including muscle and body composition. Its influence depends on the intensity of physical activity [9–14]. Foong et al. (2016) [10] reported that the intensity of physical activity had a dose–response relationship with lean mass and lower limb strength, with the largest effect on vigorous activity in community-dwelling older adults. They also reported that the duration of sedentary behavior (SB) was negatively associated with lean mass. Recently, an increasing number of studies have examined the association between intensity-specific physical activity and PhA in community-dwelling older adults in Japan or Asia [13–16]. For example, Asano et al. (2023) reported that greater time spent in moderate-to-vigorous physical activity (MVPA) was positively associated with PhA, whereas light physical activity (LPA) and SB were not [13]. However, given that the preceding studies focused on community-dwelling older adults without disabilities, the intensity of physical activity that influences the PhA in older adults requiring care due to disabilities is unclear. This focus is particularly important because older adults with disabilities are more likely to experience impaired physical function, malnutrition, and reduced physical activity in comparison to their healthier counterparts [17, 18]. A further decline in physical function frequently results in an escalation of care levels, institutionalization, and increased healthcare expenditures. Consequently, identifying this association is crucial not only for preventing further decline in their level of care but also to support the sustainability of long-term care systems.

This study aimed to clarify the relationships between PhA and SB, LPA, and MVPA, which were objectively measured using accelerometers in older adults with disabilities under the long-term care insurance system. We hypothesized that MVPA duration is positively associated with PhA in older adults with disabilities.

### Participants

**Fig. 1:**
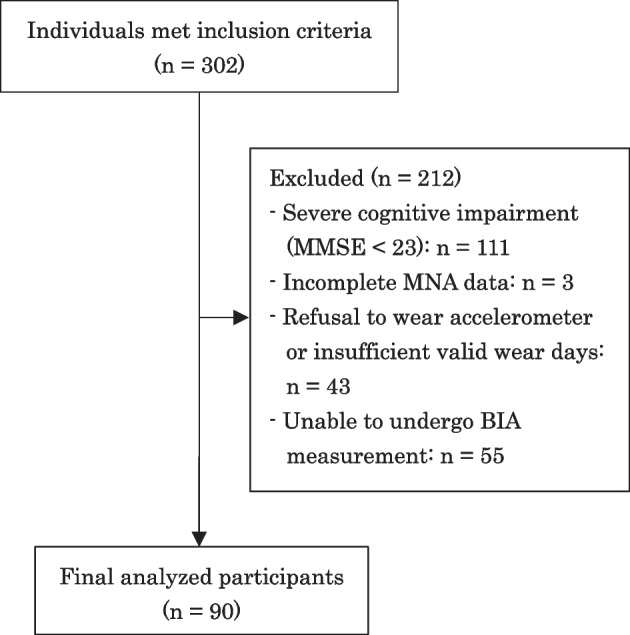
Flow diagram of participant selection. MMSE, Mini-Mental State Examination; MNA, Mini Nutritional Assessment; BIA, Bioelectrical impedance analysis

From January 2020 to April 2023, we recruited community-dwelling older adults with disabilities under the long-term care insurance system at the daycare center of Kansai Medical University Kori Hospital, Osaka, Japan. Inclusion criteria were 1) age ≧ 60 years old and 2) utilization of outpatient daycare rehabilitation services. The exclusion criteria were 1) severe cognitive impairment (Mini-Mental State Examination [MMSE] score < 23) and 2) incomplete data for any measurement. A total of 302 older adults initially met the inclusion criteria. Of these, 212 were excluded due to severe cognitive impairment (MMSE < 23; *n* = 111), incomplete data for the long form of the Mini Nutritional Assessment (MNA; *n* = 3), incomplete physical activity data because of refusal to wear the accelerometer or insufficient valid wear days (*n* = 43), and inability to undergo BIA measurement (*n* = 55) owing to pacemaker implantation, other metallic implants, or device errors. Consequently, 90 participants (39 men and 51 women) were included in the final analysis. The participant selection process is shown in Fig. 1.

Written informed consent was obtained from all participants prior to their participation. Before enrollment, the study purpose, procedures, potential benefits and risks, and the right to withdraw at any time were explained by the investigators. Participants were given the opportunity to ask questions, and only those who fully understood the information and voluntarily agreed signed the consent form. This study was approved by the Ethics Committee of Kansai Medical University (2,018,251).

### Bioelectrical impedance analysis measurements

Muscle mass and PhA were measured using a multifrequency BIA instrument (InBody S10, InBody Japan, Tokyo, Japan). Water consumption, food intake, and toileting before BIA measurement were not standardized in this study. This was because our participants were older adults with disabilities, for whom such standardization was difficult, and recent studies have shown that these factors have only minor influence [19, 20]. The participants assumed a supine position with their limbs extended, and two impedance components, R and Xc, for six different frequencies (1, 5, 50, 250, 500, and 1,000 kHz) were obtained at the body segments of the right and left upper limbs, trunk, and right and left lower limbs. Skeletal muscle mass index (kg/m^2^) was calculated by dividing ASM (the sum of the muscle mass of all four limbs) by the square of the height. Considering the importance of lower limb muscles [11], lower limb SMI and PhA were also obtained. The lower limb SMI (kg/m^2^) was calculated by combining the muscle mass of the bilateral lower limbs and dividing it by the square of the height [21, 22]. PhA (degrees) at 50 kHz were obtained as [arctangent (Xc/R) × 180°/π] for the lower limb on both sides, and the average value of both lower limbs was calculated.

### Physical activity measurements

The participants wore a triaxial accelerometer (HJA-750C, Omron Healthcare, Kyoto, Japan) on their waist for seven consecutive days, except while bathing and sleeping. We used data from at least three valid days [23], where a valid day was defined as at least 600 min/day of wear time each day. Metabolic equivalents (METs) were estimated using an internal triaxial accelerometer at 10-s intervals (epochs). Sedentary behavior was defined as METs ≦ 1.5; LPA, 1.5 to 2.9 METs; and, MVPA, METs ≧ 3.0. The durations of SB, LPA, and MVPA per day (min/day) were calculated and averaged over valid days.

### Clinical information

Information on the care or support level, which was classified into seven categories (requiring help, 1–2; long-term care level, 1–5), and that of medical history of musculoskeletal diseases, neurological diseases, heart failures, and cancers was obtained from the reports of care service managers. Nutritional status was assessed using the long form of the MNA [24].

### Statistical analysis

Continuous data are presented as mean ± standard deviation, and categorical data are presented as numbers (%). Differences between older men and women were examined using an unpaired t-test for continuous variables or Pearson χ^2^ test for categorical variables. Correlations between lower limb SMI, PhA, age, MNA score, and physical activity were examined using Pearson correlation analysis separately for men and women, because significant sex differences were found in most of the variables. Multivariable linear regression analyses were used with lower limb SMI and PhA as dependent variables. In Model 1, age, sex, and physical activity were included as independent variables. In Model 2, the MNA score and medical history (musculoskeletal diseases, neurological diseases, heart failure, and cancer) were also entered as independent variables. All statistical analyses were performed using SPSS for Windows (version 22.0; SPSS Inc., Tokyo, Japan). Statistical significance was set at p < 0.05.

## Results

The characteristics of the study participants are presented in Table 1. The mean age in men was significantly lower than that in women (*p* = 0.037). Skeletal muscle mass index, lower limb SMI and PhA were significantly higher in older men than those in older women (p < 0.05). The SB duration was significantly longer in older men than that in older women, whereas the LPA duration was significantly longer in older women than that in older men (p < 0.001). The MVPA duration was comparable between the sexes.

**Table 1:** Characteristics of the study participants

	All (*n* = 90)		Men (*n* = 39)		Women (*n* = 51)		*P*-value
Age (years)	78.7 ± 6.7		77.1 ± 6.0		79.9 ± 6.9		0.037
Height (cm)	156.2 ± 9.3		163.9 ± 6.5		150.3 ± 6.3		< 0.001
Body mass (kg)	55.6 ± 11.1		62.7 ± 10.0		50.2 ± 8.5		< 0.001
BMI (kg/m^2^)	22.7 ± 3.4		23.3 ± 3.1		22.2 ± 3.6		0.140
SMI (kg/m^2^)	6.23 ± 1.12		7.09 ± 0.97		5.56 ± 0.70		< 0.001
Lower limb SMI (kg/m^2^)	4.95 ± 0.90		5.52 ± 0.87		4.51 ± 0.63		< 0.001
Lower limb PhA (degree)	3.63 ± 0.84		3.83 ± 0.94		3.47 ± 0.73		0.046
Physical activity (min/day)							
SB	649.9 ± 195.3		732.5 ± 221.9		586.8 ± 145.2		0.001
LPA	253.8 ± 98.5		191.9 ± 73.5		301.1 ± 88.9		< 0.001
MVPA	9.1 ± 13.4		9.0 ± 12.8		9.3 ± 14.0		0.908
Care or support level (requiring help, 1–2; long-term care level, 1–5) (n)	37/ 30/ 10/ 6/ 5/ 2/ 0		17/ 11/ 5/ 2/ 3/ 1/ 0		20/ 19/ 5/ 4/ 2/ 1/ 0		0.892
Medical history, n (%)							
Musculoskeletal diseases	59 (65.6)		19 (48.7)		40 (78.4)		0.004
Neurological diseases	12 (13.3)		8 (20.5)		4 (7.8)		0.117
Heart failures	7 (7.8)		5 (12.8)		2 (3.9)		0.232
Cancers	17 (18.9)		11 (28.2)		6 (11.8)		0.060
MNA	23.0 ± 3.8		23.5 ± 3.7		22.6 ± 3.8		0.241

*BMI* body mass index, *SMI* skeletal muscle mass index, *PhA* phase angle, *SB* sedentary behavior, *LPA* light physical activity, *MVPA* moderate-to-vigorous physical activity, *MNA* Mini Nutritional Assessment

The correlation coefficients between lower limb SMI and PhA and age, MNA score, and physical activity are shown in Table 2. Lower limb SMI was significantly positively correlated with the MNA score for both men (*p* = 0.013) and women (*p* = 0.041). Lower limb PhA in men was significantly positively correlated with the MNA score (*p* = 0.046) and MVPA duration (*p* = 0.042) and that in women significantly positively correlated with LPA duration (*p* = 0.048).

**Table 2:** Correlation coefficients between lower limb SMI, PhA, age, MNA score, and physical activity

	Men (*n* = 39)				Women (*n* = 51)			
	Lower limb SMI		Lower limb PhA		Lower limb SMI		Lower limb PhA	
	r	*P* value	r	*P* value	r	*P* value	r	*P* value
Age	0.063	0.701	-0.134	0.418	-0.159	0.265	-0.213	0.134
MNA	**0.394**	**0.013**	**0.321**	**0.046**	**0.287**	**0.041**	0.198	0.164
SB	0.014	0.935	0.146	0.376	-0.044	0.759	0.056	0.695
LPA	-0.008	0.961	0.133	0.419	0.059	0.680	**0.278**	**0.048**
MVPA	0.144	0.380	**0.328**	**0.042**	-0.031	0.830	0.207	0.146

*Abbreviations*: *SMI* skeletal muscle mass index, *PhA* phase angle, *MNA* Mini-Nutritional Assessment, *SB* sedentary behavior, *LPA* light physical activity, *MVPA* moderate-to-vigorous physical activity

The factors associated with lower limb SMI on multivariable linear regression analyses are shown in Table 3. In Model 1, sex was the only variable that was significantly associated with lower limb SMI (p < 0.001). In Model 2, sex (p < 0.001) and MNA score (*p* = 0.002) were significantly associated with lower limb SMI. Further, SB, LPA, or MVPA durations were not significantly associated with lower limb SMI in either model.

**Table 3:** Factors associated with lower limb SMI on multivariable linear regression analyses

	Model 1 (R^2^ = 0.565)					Model 2 (R^2^ = 0.644)				
	β	Standardized β	95% CI		*p* value	β	Standardized β	95% CI		*p* value
(constant)	**6.012**		**(3.650, 8.375)**		** < 0.001**	**5.074**		**(2.621, 7.528)**		** < 0.001**
Age	-0.006	-0.048	(-0.032, 0.019)		0.621	-0.014	-0.103	(-0.040, 0.012)		0.298
Sex (women)	**-1.008**	**-0.561**	**(-1.410, -0.606)**		** < 0.001**	**-0.826**	**-0.460**	**(-1.240, -0.412)**		** < 0.001**
SB	0.000	-0.011	(-0.001, 0.001)		0.915	0.000	-0.052	(-0.001, 0.001)		0.614
LPA	0.000	0.010	(-0.002, 0.002)		0.932	-0.001	-0.059	(-0.003, 0.002)		0.604
MVPA	0.003	0.040	(-0.010, 0.015)		0.673	0.002	0.029	(-0.010, 0.014)		0.757
MNA						**0.071**	**0.296**	**(0.026, 0.115)**		**0.002**
Musculoskeletal disease						0.028	0.015	(-0.360, 0.416)		0.888
Neurological disease						0.062	0.024	(-0.451, 0.575)		0.811
Heart failure						0.050	0.015	(-0.551, 0.651)		0.870
Cancer						0.293	0.129	(-0.127, 0.713)		0.168

*Abbreviations*: *SMI* skeletal muscle mass index, *CI* confidence interval, *BMI* body mass index, *MNA* Mini Nutritional Assessment, *SB* sedentary behavior, *LPA* light physical activity, *MVPA* moderate-to-vigorous physical activity

The factors associated with lower limb PhA on the multivariable linear regression analyses are shown in Table 4. In Model 1, sex (*p* = 0.048) and MVPA duration (*p* = 0.034) were significantly associated with lower limb PhA. In Model 2, the MVPA duration was still significantly associated with lower limb-PhA (*p* = 0.037), whereas sex was not.

**Table 4:** Factors associated with lower limb PhA on multivariable linear regression analyses

	Model 1 (R^2^ = 0.181)					Model 2 (R^2^ = 0.272)				
	β	Standardized β	95% CI		*p* value	β	Standardized β	95% CI		*p* value
(constant)	**4.140**		**(1.710, 6.571)**		**0.001**	**3.103**		**(0.538, 5.667)**		**0.018**
Age	-0.017	-0.136	(-0.044, 0.009)		0.199	-0.013	-0.104	(-0.041, 0.014)		0.346
Sex (women)	**-0.417**	**-0.248**	**(-0.831, -0.004)**		**0.048**	-0.375	-0.222	(-0.807, 0.058)		0.089
SB	0.001	0.171	(0.000, 0.002)		0.143	0.001	0.148	(0.000, 0.002)		0.198
LPA	0.002	0.216	(0.000, 0.004)		0.096	0.002	0.206	(0.000, 0.004)		0.108
MVPA	**0.014**	**0.225**	**(0.001, 0.027)**		**0.034**	**0.014**	**0.219**	**(0.001, 0.027)**		**0.037**
MNA						0.041	0.182	(-0.006, 0.087)		0.088
Musculoskeletal disease						-0.182	-0.104	(-0.588, 0.223)		0.373
Neurological disease						0.268	0.109	(-0.268, 0.805)		0.322
Heart failure						-0.285	-0.091	(-0.913, 0.343)		0.369
Cancer						-0.251	-0.118	(-0.690, 0.188)		0.258

*Abbreviations*: *PhA* phase angle, *CI* confidence interval, *BMI* body mass index, *MNA* Mini Nutritional Assessment, *SB* sedentary behavior, *LPA* light physical activity, *MVPA* moderate-to-vigorous physical activity

## Discussion

The MVPA duration was associated with greater lower limb PhA in older adults with disabilities under the long-term care insurance system. Previous studies on individuals without disabilities [9, 13, 14, 16] reported that a longer MVPA duration was associated with a higher PhA, which supports the results of our study. Notably, our study revealed that this association was observed in older adults with disabilities under a long-term care insurance system. Older adults who require long-term care often experience malnutrition and various diseases. This study confirmed that the association between MVPA duration and lower limb PhA is independent of nutritional status or the presence of diseases such as musculoskeletal diseases, neurological diseases, heart failure, and cancers. Thus, the results are consistent with our hypothesis.

Compared with previous studies of older adults without disabilities which were conducted in populations of the same ethnicity (Japanese or Asian) [13–16], the participants in the present study exhibited longer SB duration and shorter LPA and MVPA durations. For example, Nakashima et al. [14] reported that Japanese older adults of a similar mean age (78.3 years) spent 346.3 min/day in LPA and 38.0 min/day in MVPA (SB not reported), both of which were longer than the durations observed in the present study. Furthermore, a large-scale study by Oshita et al. [25] demonstrated that, in the 75–84-year age group, the mean SMI was 7.45 kg/m^2^ for men and 6.21 kg/m^2^ for women, whereas the lower limb PhA values were 4.35° in men and 4.12° in women. In contrast, the participants in the present study exhibited lower SMI (7.09 kg/m^2^ in men and 5.56 kg/m^2^ in women) as well as lower limb PhA (3.83° in men and 3.47° in women). Taken together, these comparisons underscore the importance of demonstrating that the association between MVPA duration and lower limb PhA is evident even in older adults who have lower activity levels, reduced muscle mass, and poorer muscle quality.

A dose–response relationship exists between the intensity of physical activity and health-related outcomes, and SB duration is negatively associated with muscle mass and function [10–12]. However, SB or LPA duration was not associated with lower limb PhA after adjusting for potential confounders. This finding is consistent with the results of Asano et al. (2023) [13] and suggests that SB and LPA durations may have little influence on PhA. These results may imply that, even in older adults with disabilities and longer SB duration, lower limb PhA could be increased by ensuring a high MVPA duration.

Another unique aspect of this study is that we simultaneously measured lower limb SMI and PhA to examine their associations with intensity-specific physical activity. Notably, none of the physical activity intensities were associated with lower limb SMI in multivariable linear regression analyses. This result is inconsistent with that of a previous study by Foong et al. (2016) [10] that reported a significant association between physical activity level and lean mass in community-dwelling older adults. A possible reason for this inconsistency is the differences in the characteristics of the study participants. The participants in our study (78.7 ± 6.7 years) were older than those in study by Foong et al. (66 ± 7 years) and had disabilities and required long-term care. As the extracellular space between muscle fibers increases with age, muscle mass measures such as SMI may overestimate the actual contractile tissues in older adults [26, 27]. Therefore, the association between lower limb SMI and physical activity may have been diminished in this study. This consideration may be strengthened by the findings of Foong et al. (2016) [10] that suggested that the magnitude of the association between lean mass and physical activity diminishes with increasing age. In contrast, PhA reflects cellular health, such as cell membrane integrity and function, and it may be less affected by an increase in extracellular fluid. Therefore, PhA may be a useful measure of muscle properties sensitive to physical activity. Although lower limb SMI was not associated with physical activity, it was significantly associated with MNA after adjusting for confounders. Malnutrition is a major factor in low muscle mass or sarcopenia [28]. Our findings indicate that muscle mass is a reliable indicator of nutritional status, potentially supporting the role of physical activity in older adults who require care.

The results of this study suggest a possible association between regular MVPA and the maintenance or improvement of PhA in older adults with disabilities. However, some forms of MVPA such as strength training may not be appropriate for older adults with disabilities to include in their daily routines due to their high intensity. The mean MVPA duration of the participants in this study was only 9.1 min/day. Walking may be suitable for this population because it is a popular, highly accessible, inexpensive, and safe activity [29]. There was a close association between daily step count and daily duration of MVPA [30]. Kerr et al. (2018) [31] reported that MVPA duration in community-dwelling older adults increased via multilevel physical activity intervention, including individual counseling and self-monitoring with pedometers, group education sessions, and group walks. Koizumi et al. (2009) [29] reported that the promotion of daily physical activity using accelerometers improved the number of daily steps by 16% and the daily duration of moderate-intensity physical activity by 53% in community-dwelling older women. Further studies are needed to explore whether physical activity intervention via a walking program improves MVPA duration and PhA among older adults with disabilities.

This study has some limitations that should be considered. First, a large number of older adults were excluded, and the final sample size was small. This was because the older adults with disabilities targeted in this study were of advanced age, many had cognitive impairment, and a considerable number were unable to complete BIA or physical activity measurements. Therefore, the results of this study may not be generalizable to all older adults with disabilities. Second, this was a cross-sectional study, and the causal relationship between physical activity and PhA remains unclear. Longitudinal studies with larger sample sizes are necessary to explore whether MVPA influences future PhA changes in older adults with disabilities.

## Conclusion

Lower limb PhA, but not lower limb SMI, was associated with MVPA duration in older adults who required long-term care due to disabilities. Phase angle is gaining attention as an indicator of muscle quality that affects physical function. To maintain PhA and prevent further decline in physical function in older adults with disabilities, enhancing the duration of MVPA is needed.

## Data Availability

The data that support the ﬁndings of this study are available from the corresponding author upon reasonable request.

## Abbreviations

PhA: Phase angle
SMI: Skeletal muscle mass index
MVPA: Moderate-to-vigorous physical activity
LPA: Light physical activity
SB: Sedentary behavior
MNA: Mini-nutritional assessment
BIA: Bioelectrical impedance analysis
ASM: Appendicular skeletal muscle mass
MMSE: Mini-Mental State Examination
